# Unlocking pandemic potential: prevalence and spatial patterns of key substitutions in avian influenza H5N1 in Egyptian isolates

**DOI:** 10.1186/s12879-018-3222-6

**Published:** 2018-07-06

**Authors:** Sean G. Young, Andrew Kitchen, Ghazi Kayali, Margaret Carrel

**Affiliations:** 1 Department of Environmental and Occupational Health, University of Arkansas for Medical Sciences, Little Rock, AR USA; 2 Department of Anthropology, University of Iowa, Iowa City, IA USA; 3 Department of Epidemiology, Human Genetics, and Environmental Sciences, University of Texas Health Sciences Center, Houston, TX USA; 4 Department of Scientific Research, Human Link, Hazmieh, Lebanon; 5 Department of Geographical and Sustainability Sciences, University of Iowa, Iowa City, IA USA; 6 Department of Epidemiology, University of Iowa, Iowa City, IA USA

**Keywords:** Landscape genetics, Avian influenza, Egypt, Poultry

## Abstract

**Background:**

Avian influenza H5N1 has a high human case fatality rate, but is not yet well-adapted to human hosts. Amino acid substitutions currently circulating in avian populations may enhance viral fitness in, and thus viral adaptation to, human hosts. Substitutions which could increase the risk of a human pandemic (through changes to host specificity, virulence, replication ability, transmissibility, or drug susceptibility) are termed key substitutions (KS). Egypt represents the epicenter of human H5N1 infections, with more confirmed cases than any other country. To date, however, there have not been any spatial analyses of KS in Egypt.

**Methods:**

Using 925 viral samples of H5N1 from Egypt, we aligned protein sequences and scanned for KS. We geocoded isolates using dasymetric mapping, then carried out geospatial hot spot analyses to identify spatial clusters of high KS detection rates. KS prevalence and spatial clusters were evaluated for all detected KS, as well as when stratified by phenotypic consequence.

**Results:**

A total of 39 distinct KS were detected in the wild, including 17 not previously reported in Egypt. KS were detected in 874 samples (94.5%). Detection rates varied by viral protein with most KS observed in the surface hemagglutinin (HA) and neuraminidase (NA) proteins, as well as the interior non-structural 1 (NS1) protein. The most frequently detected KS were associated with increased viral binding to mammalian cells and virulence. Samples with high overall detection rates of KS exhibited statistically significant spatial clustering in two governorates in the northwestern Nile delta, Alexandria and Beheira.

**Conclusions:**

KS provide a possible mechanism by which avian influenza H5N1 could evolve into a pandemic candidate. With numerous KS circulating in Egypt, and non-random spatial clustering of KS detection rates, these findings suggest the need for increased surveillance in these areas.

## Background

Highly pathogenic avian influenza H5N1 has a case fatality rate approximately ten times higher than the H1N1 “Spanish Flu” pandemic of 1918, but has much lower infectivity [1, 2]. Given the high pathogenicity of the virus, an H5N1 virus capable of airborne human-to-human transmission could lead to a devastating pandemic [3–5]. Indeed, this scenario has been predicted for many years [1, 2, 6, 7]. A number of important genetic differences between avian and human strains of influenza exist, which have so far prevented H5N1 from being transmitted directly from human to human [8–11]. However, years of viral evolution in domestic poultry populations involving close contact with humans and other mammals has the potential to lead to an accumulation of mutations improving mammalian adaptation [1, 12].

Egypt has reported more confirmed human cases of avian H5N1 than any other country, and is regarded by many observers as the epicenter of human H5N1 infections [1, 13–17]. Human infections have been increasing in Egypt, and nearly all cases have been linked to direct contact with poultry [18]. The potential for direct human-poultry contact is high in Egypt. In addition to the commercial poultry industry, which supplies most of the meat consumed in the country, some 4–5 million Egyptian families raise poultry at home in backyard flocks [19, 20]. A 3-year seroprevalence study found approximately 2% of Egyptians exposed to poultry had been infected with H5N1 [21]. Contact between human and avian hosts is a precondition for virus transfer, so an environment conducive to frequent contact, such as exists in Egypt, increases the probability of a human-adapted strain emerging [22, 23].

Genetic changes in viral proteins can have phenotypic consequences that increase pandemic risk [24]. A number of specific amino acid substitutions have been implicated in host specificity, particularly changes in the hemagglutinin (HA) protein that allow the virus to bind with and infect mammalian cells [2, 25–28]. In addition, there are genetic mutations known to alter the pathogenicity of the virus, increase the replicative ability of the polymerase complexes, and grant aerosol transmissibility without reassortment [28–31]. Viral genetic changes have been identified that alter susceptibility to current anti-viral drugs such as zanamivir and oseltamivir [24, 32]. All of the above are examples of phenotypic changes resulting from amino acid substitutions, often SNPs. Some potentially dangerous mutations may simultaneously compromise viral fitness, requiring the presence of other counteracting or “permissive” mutations to restore viral fitness in order to present a threat [33]. We refer to these genetic changes (or sets of concurrent changes) that alter host specificity, virulence, replication efficiency, transmissibility, or drug susceptibility in ways that increase human risk as key substitutions (KS).

Several KS have already been detected in Egypt, from samples at least as early as 2010 [18, 34, 35]. Gain-of-function studies have suggested that as few as four or five KS may be sufficient to provide H5N1 with airborne transmission, and at least two of these have been previously identified in Egyptian isolates [5, 31, 36]. Using viral genetic sequences for H5N1 uploaded to GenBank (https://www.ncbi.nlm.nih.gov/genbank/), we performed a descriptive spatial analysis to examine the distribution of KS in Egypt [37]. A spatial analysis of KS in H5N1 influenza samples has not been performed previously, despite the importance of such information to a properly designed surveillance system and subsequent intervention efforts [13, 38]. Importantly, viruses can undergo local adaptation in response to environmental selective pressures and produce clusters of KS [39]. Areas where known KS occur with high frequency and exhibit non-random spatial clustering may indicate environments particularly conducive to the natural emergence of de novo KS.

## Methods

We obtained Egyptian H5N1 sequences collected between 2005 and 2015 from the Influenza Virus Sequence Database, hosted by the National Center for Biotechnology Information (NCBI) [40]. We extracted metadata for Egyptian samples, including geographic location information, from the full GenBank records, obtained using the Entrez Direct utilities via UNIX terminal [37, 41]. Inclusion criteria included all partial and complete protein sequences from avian hosts (both poultry and wild birds) with a geographic location more detailed than “Egypt.” A large number of H5N1 KS have been identified by the WHO Collaborating Center for Influenza Reference and Research at the CDC and are catalogued in a Genetic Changes Inventory, publicly available online (www.cdc.gov/flu/avianflu/h5n1/inventory.htm). We used this inventory in the current study as the definitive list of known KS. KS have been identified in all 10 viral proteins, including the surface proteins HA and neuraminidase (NA) responsible primarily for virus binding, cell fusion, and viral release, and the internal proteins (M1, M2, NP, NS1, NS2, PB1, PB1-F2, and PB2) involved in viral structure, replication, suppressing host antiviral response, and more [2]. We performed an initial screening for over 100 KS, ultimately testing for the presence of 39 separate substitutions.

Most of the selected KS (*n* = 29) are SNPs, such as the substitution from glutamic acid to lysine at position 627 (E627K) in the PB2 gene, shown to enhance mammalian host adaptation; two KS are amino acid deletions; and the remaining eight KS are sets of mutations (i.e., KS formed from the interaction of multiple independent substitutions), such as the dual substitutions of N200S in the NS1 gene and T47A in the NS2 gene, which together decrease host antiviral response but do not constitute KS individually. Table 1, adapted from the CDC’s Genetic Changes Inventory, lists all detected KS in Egypt, including the number of isolates in which each KS was detected, whether or not the KS had been previously reported in Egypt, and the phenotypic consequences identified in previous literature. All 39 KS are associated with increased human risk, with two partial exceptions: 1) a triple substitution of S155 N, T156A, and S223 N in the HA gene associated with both increased mammalian adaptation and reduced lethality in mice; and 2) the N66S substitution in the PB1-F2 gene associated with increased virulence and increased host antivirus response [42–45]. We selected five phenotypic consequences groups (PCGs) using the CDC’s Inventory to group KS by the resultant changes in viral characteristics (i.e. phenotypic consequences): PCG1 – host specificity, PCG2 – virulence, PCG3 – replication efficiency, PCG4 – transmissibility, and PCG5 – antiviral susceptibility. These phenotypic consequences were identified by the CDC as “signal[ing] adaptation to mammalian species or alter[ing] susceptibility to existing antivirals” thereby rendering the virus more dangerous to humans [46].

**Table 1:** Detected KS in Egypt with phenotypic consequences and references, adapted from CDC’s Inventory

Protein	Amino Acid Change(s)	Detections in Egypt	Previously Reported in Egypt	Phenotypic Consequences
HA	D94N	738	Yes	Increased viral binding to alpha,2–6; enhanced virus fusion [59]
	S133A	40	Yes	Increased pseudovirus binding to alpha,2–6 [77]
	A134V	1	Yes	Increased infectivity in SIAT Cells [78, 79]
	G139R	7	No	Increased virus binding to alpha,2–6 [80]
	S155N	470	Yes	Increased virus binding to alpha,2–6 [81]
	T156A	656	Yes	Increased virus binding to alpha,2–6 and increased transmission in guinea pigs [81, 82]
	N182K	3	Yes	Increased virus binding to alpha,2–6 [78, 80, 83]
	E186G	1	No	Increased virus binding to alpha,2–6 [25]
	T188I	17	Yes	Increased pseudovirus binding to alpha,2–6 [77]
	K189R	722	Yes	Increased virus binding to alpha,2–6 [81]
	Q192R/H	11	Yes	Increased virus binding to alpha,2–6 [25, 80, 84]
	N193K	1	No	Increased virus binding to alpha,2–6 [80]
	V210I	12	Yes	Increased virus binding to alpha,2–6 [84]
	S223N	6	Yes	Increased virus binding to alpha,2–6 [25, 78, 83, 85, 86]
	P235S	733	Yes	Increased virus binding to alpha,2–6 [84]
	E75K, S123P	1	No	Increased virus binding to alpha,2–6 [80]
	L129-, I151T	453	Yes	Increased virus binding to alpha,2–6 [84, 87]
	S133A, T188I	1	No	Increased pseudovirus binding to alpha,2–6 [77]
	S155N, T156A	359	Yes	Increased virus binding to alpha,2–6 [81]
	S155N, T156A, S223N	3	No	Increased virus binding to alpha,2–6; reduced lethality and systemic spread in mice [42]
	T156A, S223N	6	No	Increased virus binding to alpha,2–6 [81]
M1	N30D	99	Yes	Increased virulence in mice [88]
	T215A	100	No	Increased virulence in mice [88]
M2	V27A	1	No	Reduced susceptibility to amantadine and rimantadine [89–92]
	S31N/G	53	Yes	Reduced susceptibility to amantadine and rimantadine [89, 90, 93–97]
NA	49–68 deletion	366	Yes	Enhanced virulence in mice [98, 99]
	I97V	1	No	Reduced susceptibility to oseltamivir [100–102]
	I203M/V/L/K/R	1	No	Reduced susceptibility to oseltamivir [103–107]
	H254Y/R	379	No	Reduced susceptibility to oseltamivir and peramivir [103, 108–112]
	N275S	4	Yes	Reduced susceptibility to oseltamivir [106, 108, 109, 113–115]
NS1	P42S	118	Yes	Increased virulence in mice [116]
	80–84 deletion	128	Yes	Increased virulence in mice [117, 118]
	L98F	111	No	Increased virulence in mice [119, 120]
	I101M	118	No	Increased virulence in mice [119, 120]
NS1 & NS2	N200S (NS1), T47A (NS2)	38	No	Decreased antiviral response in host [121]
PB1-F2	N66S	1	Yes	Increased virulence, replication efficiency, and antivirus response in mice [43–45]
PB2	E627K	94	Yes	Increased replication efficiency in cell culture and enhanced virulence in mice; enhanced polymerase activity and mammalian host adaptation; transmissible among ferrets [29, 31, 122–131]
	D701N	1	No	Enhanced replication efficiency, increased virulence and transmission in guinea pigs; mammalian host adaptation; increased virulence in mice [27, 82, 127, 132]
	L89V, G309D, T339L, R477G, I495V, L627E, A676T	1	No	Enhanced polymerase activity and increased virulence in mice [60]

We aligned the sequences with the A/Vietnam/1203/2004 virus, except for the NA and NS1 proteins, which we aligned with A/goose/Guangdong/1/1996, for consistent amino acid numbering with the CDC’s Inventory, and then tested for the presence of each selected KS. H5N1 samples in GenBank varied in the number of proteins for which full sequence data were available. This variation occurs when researchers choose not to sequence certain proteins or fail to upload sequences to GenBank due to quality concerns, such as unconfirmed frame shifts. As not all proteins were present in GenBank for each virus sample, the specific number of KS tested for varied between samples. Detection rates by viral sample were calculated as the number of KS detected divided by the number of KS tested for, which varied according to the number of proteins sequenced for each isolate. Results were summarized by viral protein, sample year, and by PCG.

Geospatial hot spot analyses have been used to help target areas with a disproportionate burden of disease [47, 48]. We employed the Getis-Ord Gi* statistic to identify statistically significant hot spots, or spatial clusters of elevated rates of KS detection [49]. The Getis-Ord Gi* has been shown to be effective even with low incidence rates [49, 50]. For a hot spot to be detected, a location with a high value (e.g., many KS detected) must have neighbors with high values. Neighbors are determined in one of two ways. First, because the scale at which clustering occurs is not known a priori, we used Incremental Spatial Autocorrelation to identify a statistically significant peak distance at which clustering across the whole study area was most pronounced [51–53]. This distance was then used to select neighbors during hot spot analyses. If no peak was detected, an average distance was calculated to yield thirty neighbors. The local sum for a feature and its neighbors is compared proportionally to the sum of all features, then a z-score is calculated to determine when this difference is too large to be the result of random chance. Statistical significance is adjusted to account for multiple testing and spatial dependence using Benjamini’s and Hochberg’s false detection rate (FDR) correction, which reduces the critical *p*-value thresholds as a function of the number of input features and the neighborhood structure used [54, 55].

**Fig. 1:**
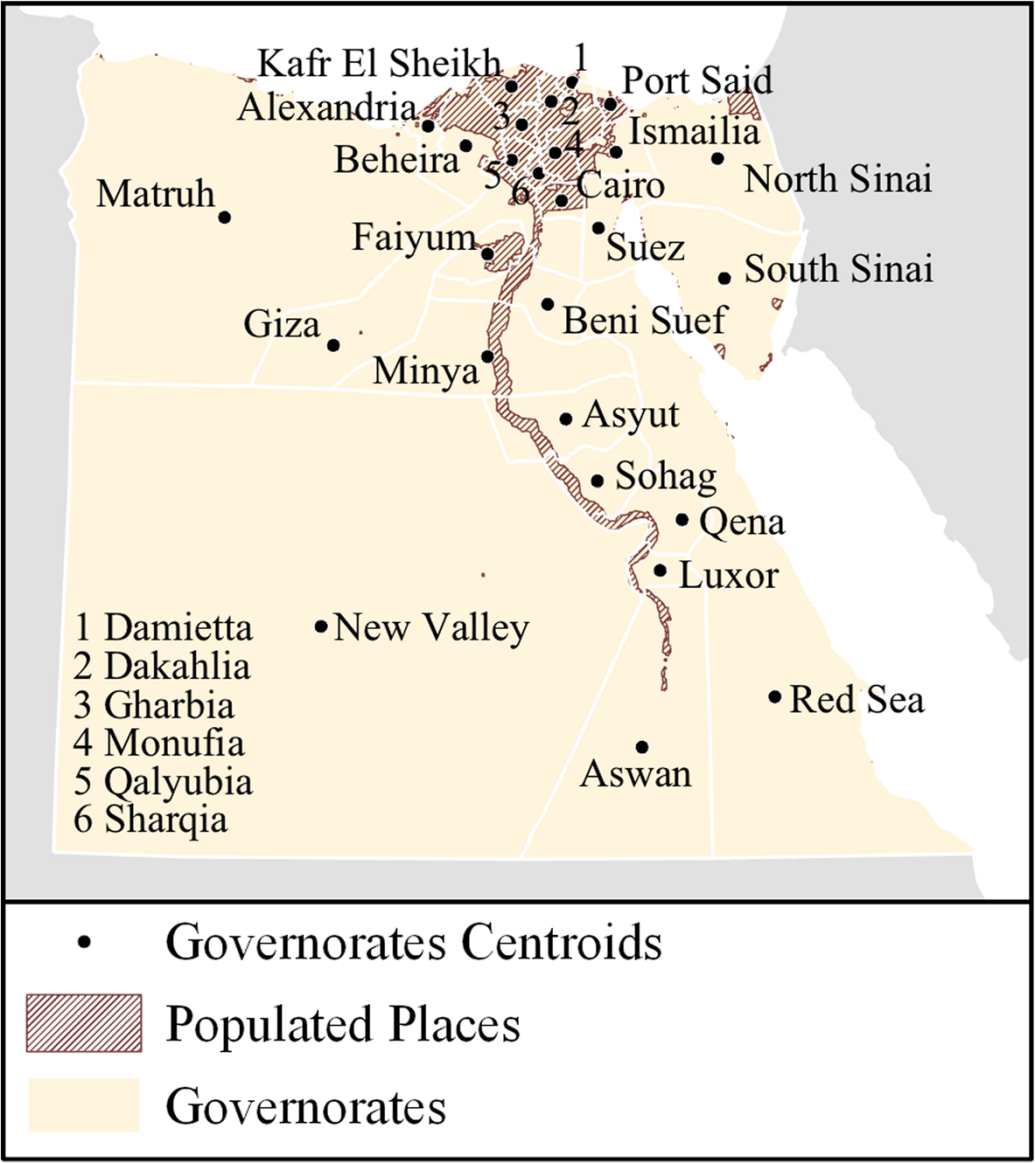
Locations for which spatial clusters of high KS detection rates were evaluated. Governorate centroids and boundaries shown, with dasymetrically-adjusted populated places shown with hatching

The majority of H5N1 samples from Egypt did not include geographic information below the governorate level to allow hot spot detection, consequently dasymetric adjustment was employed to approximate sample locations within governorates [56]. We randomly distributed point locations only within populated places, as identified using the 2010 population density estimates from worldpop.org with a minimum density value of 10 persons per square kilometer [57]. This removed large uninhabited portions of many governorates from the analysis. Acknowledging the bias introduced by estimating point locations, the random distribution of points was repeated 10,000 times, with hot spot analysis performed after each iteration. Only statistically significant hot spots (*p* ≤ 0.05) were kept. We summarized results for each governorate by counting the total number of iterations in which at least one hot spot was detected. We considered governorates with 9500 or more detections to be probable hot spots. For comparison, we also performed a single hot spot analysis using each governorate’s geometric centroid, consistent with prior phylogeographic studies of influenza in Egypt [16, 58]. Figure 1 shows the sets of locations for which spatial clusters of high KS detection rates were evaluated.

## Results

**Fig. 2:**
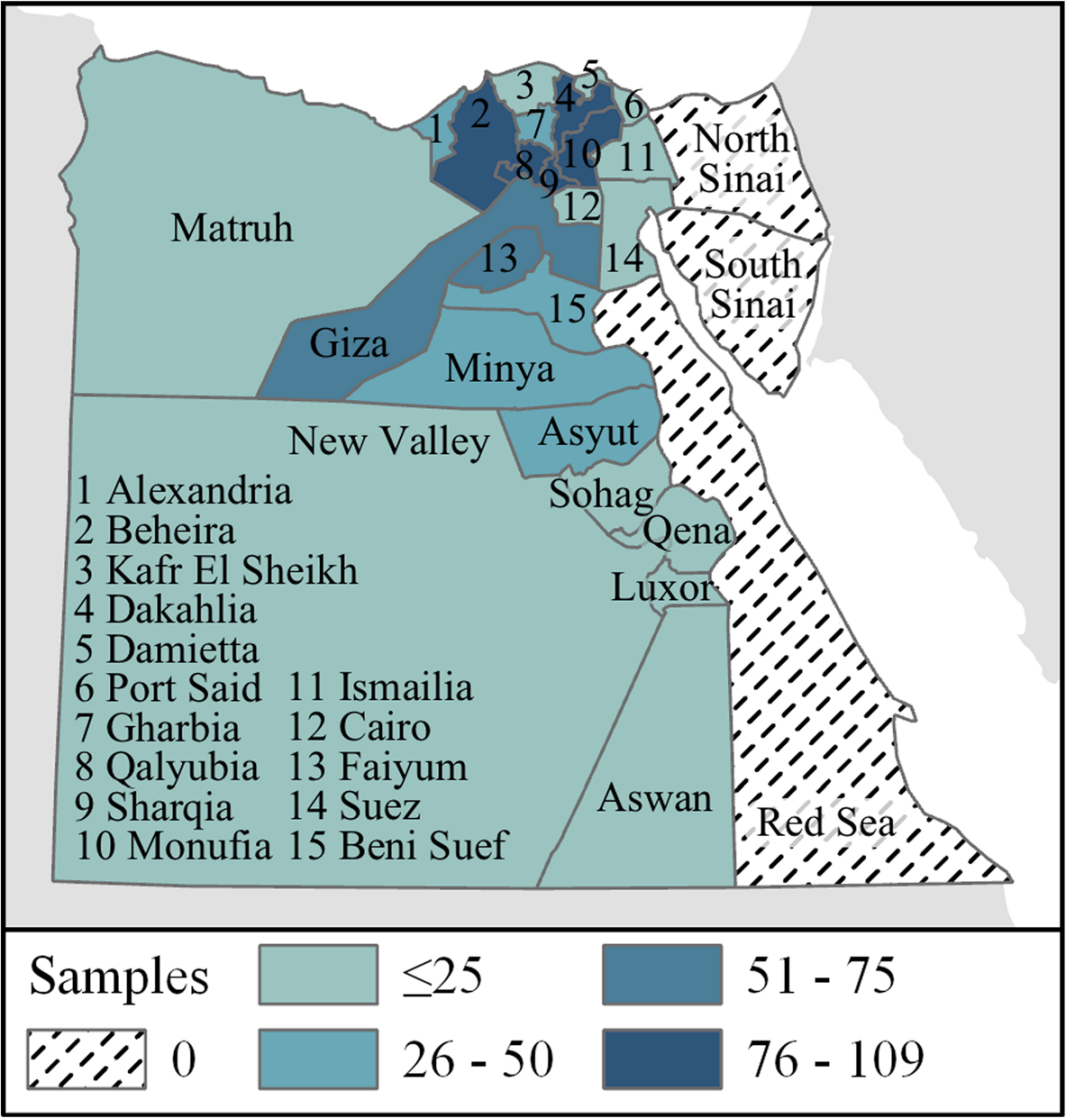
Counts of viral samples that met inclusion criteria by governorate

From 2006 to 2015, 925 H5N1 samples with at least one sequenced gene segment and a geographic location more detailed than “Egypt” were listed in GenBank. Of these, 874 samples contained at least one KS (94.5%), with an average of 3 PCGs represented. The majority of KS-positive samples (584/925) were located in Lower Egypt, particularly in the governorates of Alexandria (*n* = 50), Beheira (*n* = 96), Monufia (*n* = 90), Qalyubia (*n* = 109), Dakahlia (*n* = 82), and Sharqia (*n* = 88) (see Fig. 2).

The most commonly sequenced protein was HA (*n* = 825), followed by NA (*n* = 381); other viral proteins were much less frequent in the GenBank data. Detection rates varied by protein with the NA and NS1 proteins having one or more KS present in 100% of samples tested, while the PB1-F2 protein had only a single detected KS out of 88 samples tested. The average number of KS tested for was 22.6 per sample, with an average of 6.4 KS detected, giving an overall mean detection rate of 0.28. A total of 39 KS were detected at least once in H5N1 samples from Egypt. Table 2 summarizes the detected KS by protein and includes the proportion of samples in which KS were present.

**Table 2:** Summary of KS detections by viral protein

Protein	# of KS	# of Samples with KS Detected/Total Samples (%)
HA	21	789/825 (95.6)
M1	2	100/112 (89.3)
M2	2	54/103 (52.4)
NA	5	381/381 (100)
NS1	4	130/130 (100)
NS2	1	38/108 (35.2)
PB1-F2	1	1/88 (1.1)
PB2	3	94/116 (81)
Total^a^	39	874/925 (94.5)

^a^Many samples included multiple protein sequences, so the total number of samples is smaller than the sum of the individual protein sequences would suggest

**Fig. 3:**
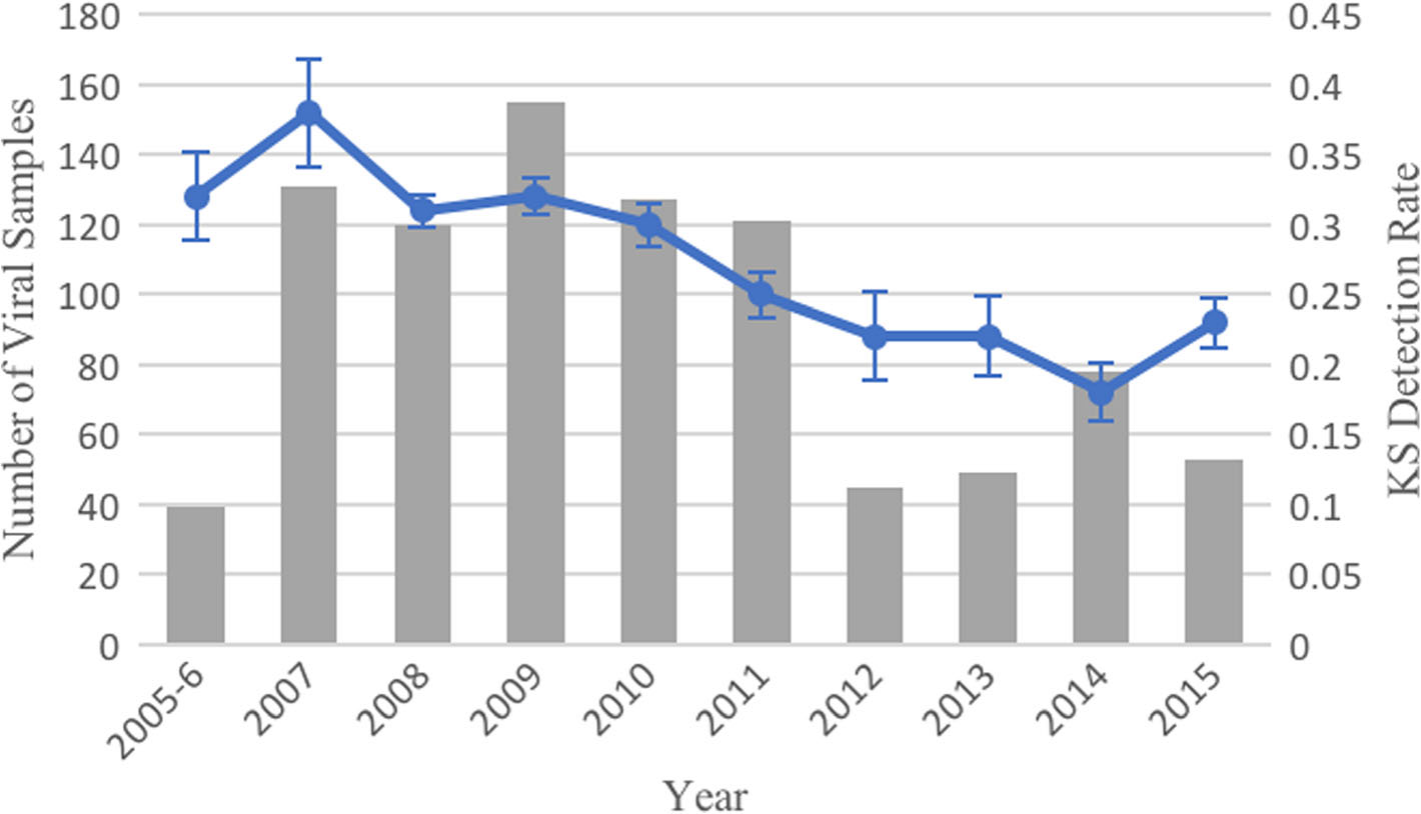
Temporal distribution of viral samples and KS detection rates by year

Although H5N1 was first reported in Egypt in 2006, 2 positive viral samples were collected in December 2005. The number of isolates uploaded to GenBank with governorate location information rose above 100 each year in 2007–2010, but dropped after 2011. Similarly, the average number of proteins sequenced per virus ranged from a high of 2.7 in 2008 to a low of 1.3 in 2014. Between 2005 and 2015, mean detection rates by year (total KS detected per year divided by total KS tested for in that year) ranged from a high of 0.38 in 2007 to a low of 0.18 in 2014. Figure 3 summarizes the temporal distribution of detected KS by year, with the 95% margin of error for KS detection rates shown.

The single most commonly detected KS was the change in the HA protein at amino acid site 94 from aspartic acid to asparagine, associated with increased virus binding to human sialic acid receptors [59]. KS were grouped according to phenotypic consequences as identified in the CDC’s Inventory. Among the detected KS were several not previously reported in Egypt (*n* = 17), representing all 5 PCGs and including 5 of the 8 detected KS associated with antiviral susceptibility. One KS not previously reported in Egypt was a set of 7 amino acid substitutions in the PB2 gene, associated with both PCG 2 and PCG 3 [60]. For a full list of detected KS with phenotypic consequences, see Table 1. The number of viral samples with one or more detected KS in each group as well as mean detection rates by PCG are summarized in Table 3.

**Table 3:** Summary of detected KS by phenotypic consequences groups (PCG)

Phenotypic Consequences Group (PCG)	KS in PCG	Samples w/ KS Detected	Mean Detection Rate
PCG1: Host Specificity	23	800/836	0.26
PCG2: Virulence	13	851/918	0.58
PCG3: Replication	5	98/846	0.08
PCG4: Transmissibility	3	704/836	0.71
PCG5: Antiviral Susceptibility	8	400/446	0.25

## Results of hot spot analyses

**Fig. 4:**
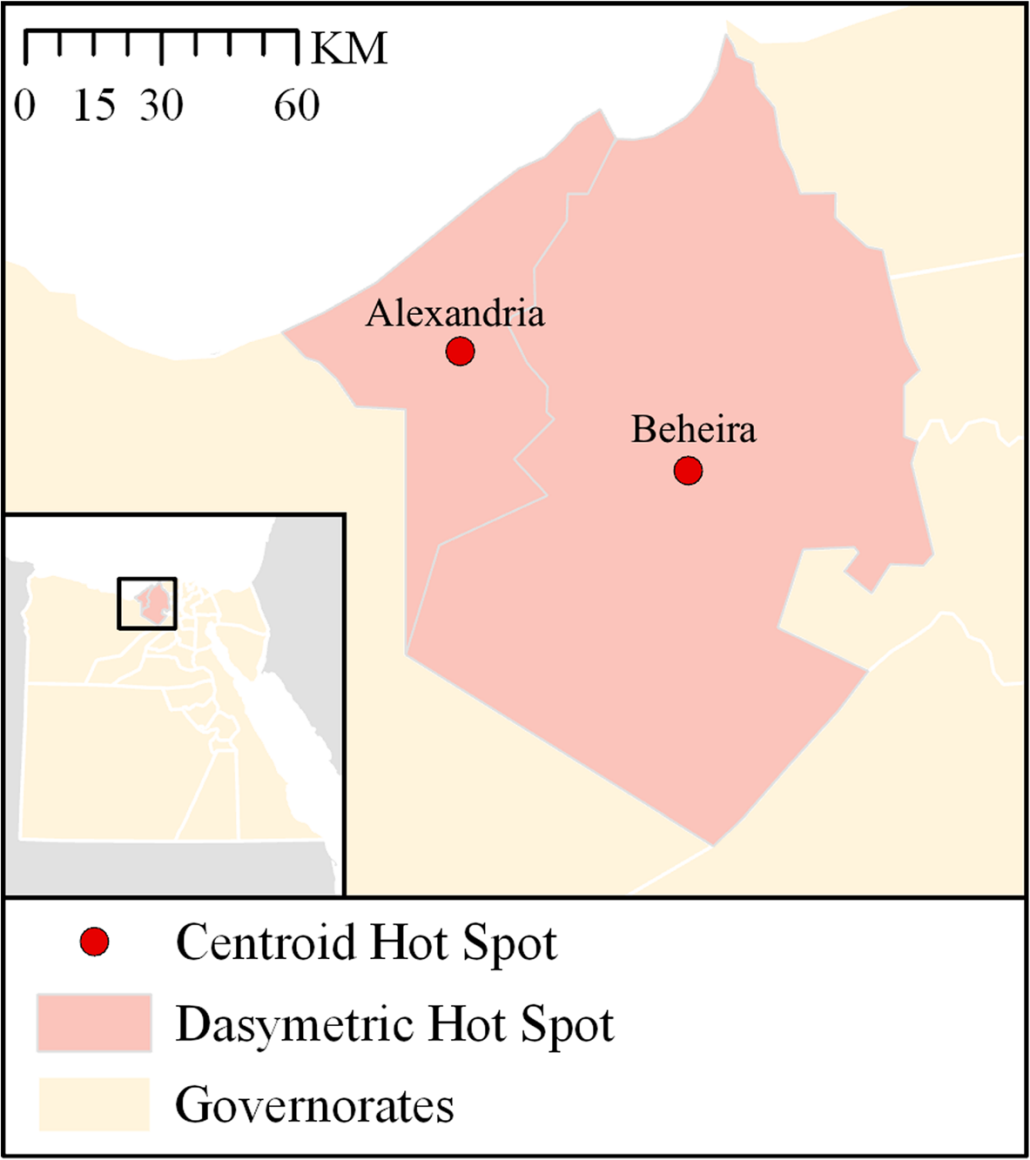
Summary of hot spot analyses for overall KS detection rates. The governorates of Alexandria and Beheira were the only locations identified as containing probable hot spots using both dasymetric adjustment (> 9500 iterations) and governorate centroids

Two governorates contained probable hot spots of overall KS detection rates using both governorate centroids (single run) and dasymetrically-adjusted governorate boundaries (> 9500 iterations): Alexandria and Beheira. These two governorates are immediate neighbors in the northwestern portion of the Nile River Delta (see Fig. 4). No other governorates were identified as hot spots using centroids, and no other governorates exceeded the threshold of 9500 iterations using dasymetric adjustment.

**Fig. 5:**
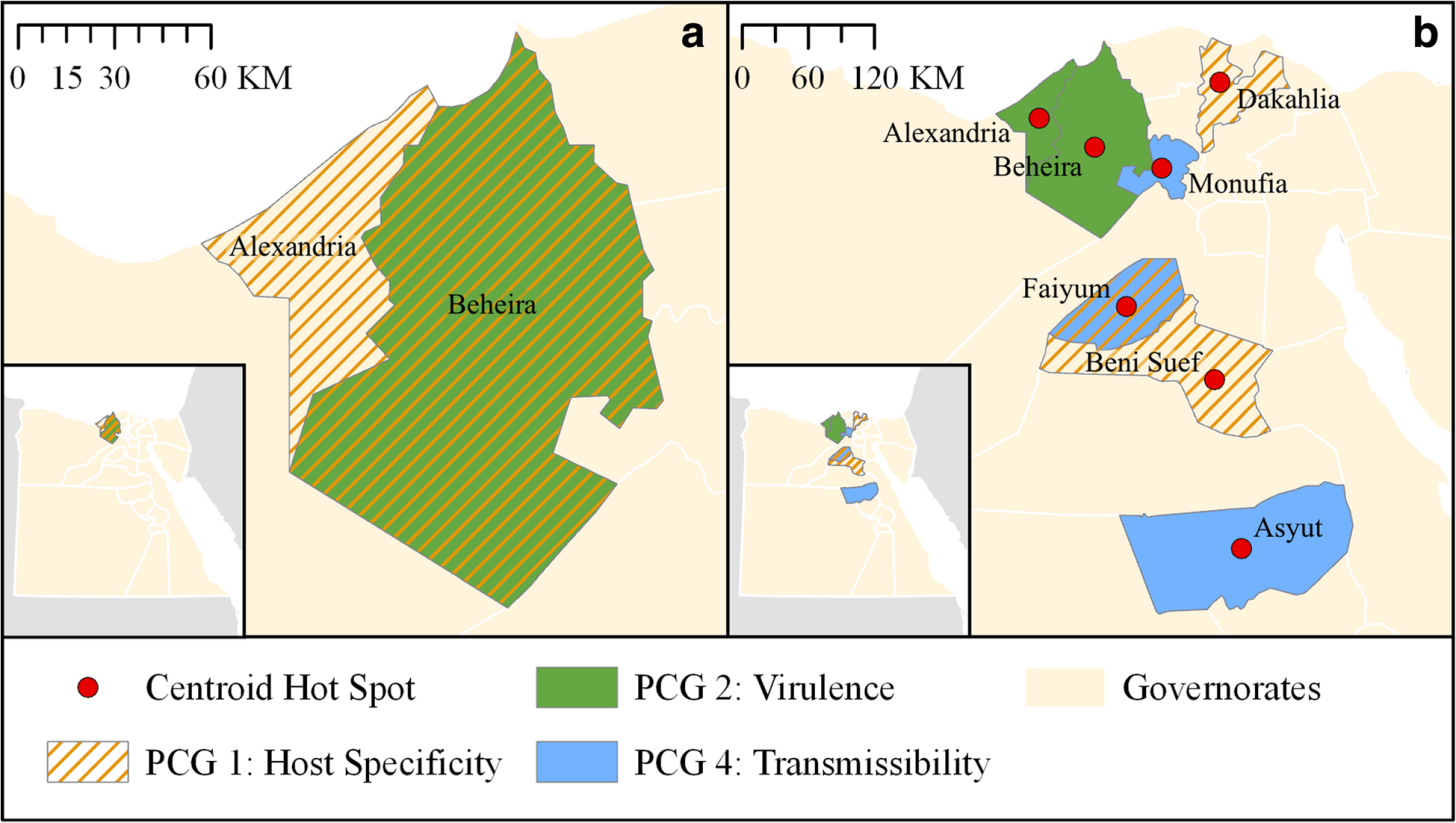
Summary of hot spot analyses for KS detection rates by PCG, using (**a**) dasymetrically-adjusted sample locations (> 9500 iterations), and (**b**) governorate centroids

Geospatial hot spots were also evaluated for individual PCGs, using a *p*-value cutoff of 0.05 to evaluate statistical significance. We detected probable hot spots based upon dasymetric adjustment for PCG1 in Alexandria and Beheira and for PCG2 only in Beheira, however the remaining PCGs falling below the 9500 iterations threshold. Results were not consistent between centroid and dasymetrically-adjusted analyses. Using a single iteration with all samples placed at governorate centroids, hot spots were found for PCG1 in Beni Suef, Dakahlia, and Faiyum; for PCG2 in Alexandria and Beheira; and for PCG4 in Asyut, Faiyum, and Monufia; while PCG3 and PCG5 again did not contain any hot spots (see Fig. 5). The number of iterations in which probable hot spots were detected using dasymetric adjustment, as well as detections using centroids, are shown in Table 4.

**Table 4:** Results of hot spots analyses for overall KS detection rates and by PCG

Governorate	Overall KS	PCG1	PCG2	PCG3	PCG4	PCG5
Alexandria	10000^a^	9927	9187^a^	17	35	2055
Aswan	0	115	0	0	132	0
Asyut	0	1	0	0	7891^a^	0
Beheira	10000^a^	9909	9778^a^	1483	2025	5304
Beni Suef	0	4533^a^	1	3	2918	0
Cairo	4	0	10	0	1	0
Dakahlia	2	677^a^	103	51	658	9
Damietta	0	9	1	26	24	0
Faiyum	2	5405^a^	0	632	8490^a^	3
Gharbia	6955	4843	3649	422	1971	3092
Giza	143	217	133	52	477	0
Ismailia	0	0	0	0	1	1
Kafr El Sheikh	6649	5308	3333	920	1700	2143
Luxor	0	83	0	0	471	0
Matruh	326	184	62	0	0	49
Minya	0	124	0	0	1214	0
Monufia	2460	942	1484	557	1738^a^	310
New Valley	0	6	0	0	200	0
North Sinai	–	–	–	–	–	–
Port Said	0	0	0	22	0	5
Qalyubia	0	0	2	0	0	0
Qena	0	111	0	0	357	0
Red Sea	–	–	–	–	–	–
Sharqia	0	0	0	16	7	0
Sohag	0	1467	0	0	1692	0
South Sinai	–	–	–	–	–	–
Suez	0	1	0	0	3	0

The number of iterations (out of 10,000) in which statistically significant hot spots were detected using dasymetric adjustment is shown for each governorate, while an ^a^indicates hot spots detected in a single run using governorate centroids

## Discussion

This work represents the first spatial analysis of KS in H5N1 avian influenza virus in Egypt, and the first use of geospatial hot spot analysis for influenza KS. The majority of viral samples (874/925) contained one or more KS, with 39 distinct KS detected including from each of the five PCGs. The circulation of so many KS increases the probability of recombination and reassortment producing viral genomes with combinations of KS that prove to be well-adapted to infect, replicate in, and transmit between humans.

Samples with governorate location information uploaded to GenBank varied from year to year, with a low of 39 in 2005–6 and a high in 2009 with 155 isolates, however the sample sizes dropped after 2011. It is not entirely clear why fewer isolates from Egypt are being uploaded to GenBank than previously, although political turmoil and social unrest in the wake of the 2011 revolution are likely responsible. Detection rates of KS also varied, trending down during the study period from a high of 0.38 in 2007 to a low of 0.18 in 2014 (see Fig. 3). This decline is likely driven a smaller number of protein sequences per sample being uploaded in later years of the study period, with the HA gene being the most commonly uploaded. HA sequences tend to have lower detection rates due to the larger number of known HA KS being tested for. The problem of outdated or incomplete reporting of avian influenza is not new in the country, and several studies suggest the true burden of disease in Egypt is severely underestimated [3, 61]. Given the inefficiency in past years of vaccination efforts in the country, there is a pressing need for increased biosecurity and public health education to help limit direct contact between humans and poultry [62–64]. Due to limited resources, the ability to focus such campaigns in regions of greatest need is one of the primary contributions of this work.

Spatial clusters of overall KS detection rates were identified using hot spot analyses in the northwestern portion of the Nile River delta region in Egypt, specifically within the governorates of Alexandria and Beheira. Hot spots were also identified for individual PCGs, however the reduced sample sizes resulting from splitting the dataset into PCGs increased the variability. This can be observed in the differences between the results using dasymetrically-adjusted locations and those using centroids. For example, hot spots were detected using centroids in Beni Suef, Dakahlia, and Monufia governorates for various PCGs, despite these governorates containing hot spots in less than half of the iterations using the dasymetrically-adjusted locations. Since both centroids and dasymetrically-adjusted locations are estimates, the latter method is preferable as its iterative nature (i.e., 10,000 replicate analyses) provides an indication of the reliability of our estimates and does not assume all samples were collected at the geometric centroid of the governorate. Using dasymetrically-adjusted locations, spatial clusters of KS detection rates were identified only in Alexandria and Beheira for KS related to host specificity and virulence (PCG1 and 2).

The city of Alexandria is the second largest city in Egypt and an important seaport, as well as a popular tourist destination. The neighboring governorate of Beheira is located between Alexandria and Cairo, experiencing substantial transport between those two urban centers. A study in Beheira in 2015 claimed the majority of the population was involved in raising poultry, and found most poultry growers demonstrated poor practices with regards to avian influenza prevention and control [65]. Spatial clustering of high rates of KS in this area suggests increased surveillance is warranted. It is not clear if the detected KS represent de novo mutations in Egypt, or are being introduced from outside the country. The identification of Alexandria and Beheira as a cluster of KS detections may suggest the latter due to their location on the Black Sea/Mediterranean flyway, but more work is required to determine this with confidence [66–69]. If the majority of detected KS are the result of viral introductions, it might partially explain the observed spatial patterns, including the relatively low rates in and around Cairo.

A limitation of this analysis was that in most governorates sample sizes were too small to allow for time series analyses of detected hot spots, therefore spatial clusters were assumed to be consistent over the 10-year study period. We were also limited by the lack of fine-grain geographic location information for influenza sequences on GenBank, which were the primary source of data for this study. Recognized by previous researchers as an important shortcoming to the otherwise incredibly useful datasets available through GenBank, the lack of consistency and infrequent inclusion of sub-governorate location information required the use of approximation methods which introduced uncertainty into the analyses [70–72]. Previous spatial investigations of Egyptian isolates relied solely on geographic centroids of governorates, or even the country [16, 58, 73–75]. This and many other studies focused on the spatial dynamics of genetic data would benefit immensely from improved geographic resolution in GenBank records. We call on researchers uploading genetic data to GenBank and similar repositories to include as much geographic detail in the metadata as possible, while respecting privacy considerations when appropriate. Sampling bias, both in terms of geographic locations chosen for sample collection and in terms of genes selected for sequencing, was a challenge for this study as with all secondary analyses. It is difficult to know to what extent such bias introduced uncertainty into the current analysis. While sampling would appear to be concentrated in the most densely populated areas of the country (see Figs. 1 and 2), it is unclear from the information available in GenBank what sampling strategies were employed or why full genome sequences were not collected or cataloged. Another challenge was that at the time of this writing, the CDC’s H5N1 Genetic Changes Inventory had not been updated since June 26, 2012. This corresponds with the publication of two papers detailing gain-of-function experiments in which KS allowing avian H5N1 transmission between mammals were identified and described, leading to concerns about public dissemination of KS information [31, 36, 76]. Despite the recent lifting of the NIH’s funding pause on gain-of-function studies in December 2017, no updates to the June 2012 edition of the inventory were published prior to this study.

## Conclusions

This work provides a descriptive baseline for the distribution and spatial clustering of KS in Egypt, from which future studies can be compared. High overall detection rates of KS were found to cluster spatially in the northwestern portion of the Nile River delta. Large numbers of KS were detected with a range of associated phenotypic consequences, which raise concerns of a pandemic strain developing in the region. Better geographic data and improved genetic surveillance are necessary to properly evaluate and monitor this potential threat to global public health.

## Abbreviations

HA: Hemagglutinin
KS: Key substitutions
MP: Matrix protein
NA: Neuraminidase
NP: Nucleoprotein
NS: Non-structural protein
PA: RNA polymerase subunit
PB1: RNA polymerase subunit
PB2: RNA polymerase subunit
PCG: Phenotypic Consequence Group
